# miR-34a, miR-424 and miR-513 inhibit MMSET expression to repress endometrial cancer cell invasion and sphere formation

**DOI:** 10.18632/oncotarget.25298

**Published:** 2018-05-01

**Authors:** Peixin Dong, Ying Xiong, Junming Yue, Sharon J.B. Hanley, Hidemichi Watari

**Affiliations:** ^1^ Department of Obstetrics and Gynecology, Hokkaido University School of Medicine, Hokkaido University, Sapporo, Japan; ^2^ Department of Gynecology, State Key Laboratory of Oncology in South China, Sun Yat-sen University Cancer Center, Guangzhou, P. R. China; ^3^ Department of Pathology and Laboratory Medicine, University of Tennessee Health Science Center, Memphis, TN, USA; ^4^ Center for Cancer Research, University of Tennessee Health Science Center, Memphis, TN, USA; ^5^ Department of Women’s Health Educational System, Hokkaido University School of Medicine, Hokkaido University, Sapporo, Japan

**Keywords:** microRNA-34a, microRNA-424, microRNA-513, MMSET, endometrial cancer metastasis

## Abstract

Although the oncogene MMSET (also known as NSD2 or WHSC1) has an essential role in malignancies, its impact on human endometrial cancer (EC) metastasis and the molecular mechanism of MMSET regulation are largely unknown. We report that MMSET was markedly upregulated in EC cell lines and EC tissues, and was significantly associated with poor survival in EC. MMSET overexpression greatly promoted EC cell invasion and sphere formation, whereas inhibition of MMSET reduced EC cell invasion and sphere formation. Importantly, Twist1 was required for MMSET-induced EC cell invasion and sphere formation. Moreover, we demonstrate that miR-34a, miR-424 and miR-513 directly modulate MMSET expression to attenuate the invasion and sphere formation capacity of EC cells. miR-34a, miR-424 and miR-513 were down-regulated in EC compared with normal tissue, and reduced expression of miR-34a, miR-424 and miR-513 was clinically associated with a poorer prognosis in EC patients. Furthermore, specific inhibition of MMET with BIX-01294 led to decreased EC cell invasion and impaired sphere formation. These findings suggest a pro-metastatic role for MMSET in EC and reveal that the repression of miR-34a, miR-424 and miR-513 contributes to the overexpression of MMSET during EC metastasis.

## INTRODUCTION

Metastasis causes > 90% of cancer-related deaths, however our understanding of the molecular mechanisms that regulate endometrial cancer (EC) metastasis remains limited. Epithelial-to-mesenchymal transition (EMT) is a critical step in promoting the acquisition of invasive and stem cell-like properties of cancer cells [1–3]. During EMT, tumor cells lose the epithelial morphology and acquire a more migratory mesenchymal-like phenotype, through downregulation of epithelial markers and upregulation of mesenchymal markers [2, 3]. EMT can be induced by several transcription factors including Twist1, which repress E-cadherin expression and increase levels of Vimentin. Elevated levels of Twist1 are associated with increased metastasis and poor survival in EC patients [4].

Epigenetic alterations play a novel role in the regulation of EMT and metastasis [5–7]. Multiple myeloma SET domain (MMSET, also known as NSD2 or WHSC1) catalyzes the dimethylation of lysine 36 on histone H3 (H3K36me2), resulting in aberrantly high global levels of H3K36me2, a mark associated with active transcription [8–10]. MMSET epigenetically activates Twist1 to promote EMT in prostate cancer [11]. MMSET is upregulated in EC through unknown mechanisms, and its overexpression has been correlated with higher grade, advanced stage and poorer patient survival [12]. MicroRNAs (miRNAs) serve as a class of oncogenes or tumor suppressors by binding to the 3′-untranslated region (UTR) of target mRNAs. However, little is known regarding the function of MMSET and the mechanisms underlying the regulation of its expression in EC.

We show that MMSET is a tumor promoter in EC, and the loss of miR-34a, miR-424 and miR-513 contributes to the overexpression of MMSET and aggressive phenotypes of EC cells.

## RESULTS

### MMSET upregulation promotes invasion and sphere formation in EC cells

**Figure 1:**
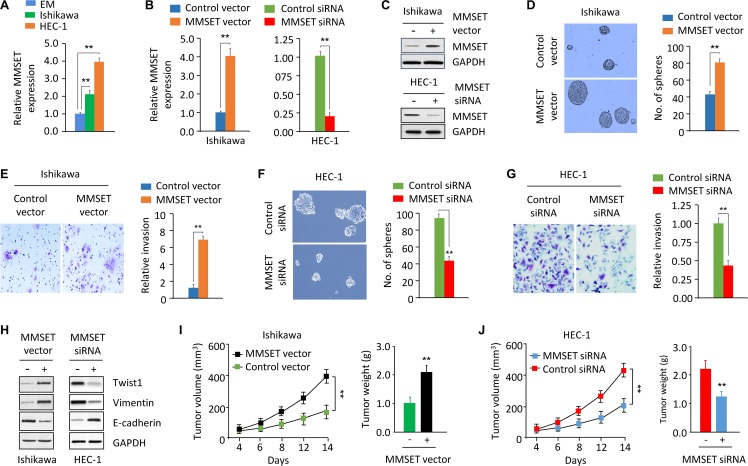
MMSET upregulation promotes sphere formation and invasion in EC cells (**A**) qPCR analysis of *MMSET* mRNA expression in the immortalized human endometrial epithelial EM cell line and EC cell lines. (**B** and **C**) qPCR analysis and western blotting analysis of *MMSET* expression in Ishikawa cells after transient overexpression of MMSET, and in HEC-1 cells after transient knockdown of MMSET. (**D** and **E**) Sphere formation (D) and invasion (E) in Ishikawa cells after transient overexpression of MMSET. (**F** and **G**) Sphere formation (F) and invasion (G) in HEC-1 cells after transient knockdown of MMSET. (**H**) Western blotting analysis of indicated proteins in Ishikawa and HEC-1 cells after overexpression or knockdown of MMSET. (**I**) Ishikawa cells were transfected with MMSET expression vector or the control vector in culture plates. 48 hours later, MMSET-overexpressing Ishikawa cells were injected into nude mice. Tumor volume and weight were measured. (**J**) HEC-1 cells were transfected with MMSET siRNA or the control siRNA in culture plates. 48 hours later, MMSET-knockdown HEC-1 cells were injected into nude mice. Tumor volume and weight were shown. ^**^P < 0.01.

We first detected *MMSET* mRNA expression in the immortalized human endometrial epithelial EM cells and in two EC cell lines (Ishikawa and HEC-1) using qPCR assay. When we compared mRNA expression between the EM and EC cells, we identified the upregulation of *MMSET* in the EC cell lines (Figure 1A). Interestingly, the aggressive EC line HEC-1 endogenously expresses high *MMSET* levels, but the less invasive EC cell line Ishikawa show low levels of *MMSET* mRNA (Figure 1A). Therefore, we transiently overexpressed MMSET by transfecting Ishikawa cells with MMSET expression vector (Figure 1B and 1C) and performed the tumor sphere formation assay. Our results showed that MMSET-transfected Ishikawa cells formed more tumor spheres with higher cell content compared with the spheres formed by control cells (Figure 1D). Furthermore, MMSET overexpression greatly promoted the invasion capability of Ishikawa cells (Figure 1E). We also explored the impact of MMSET silencing on sphere formation and invasion of HEC-1 cells by transiently knocking down MMSET expression with siRNA (Figure 1B and 1C). MMSET knockdown markedly suppressed sphere formation and invasion of HEC-1 cells (Figure 1F and 1G), suggesting that MMSET overexpression promotes the metastatic capability of EC cells *in vitro*.

To directly assess whether MMSET affects tumor formation *in vivo*, we injected MMSET-overexpressing Ishikawa cells or MMSET-knockdown HEC-1 cells into nude mice. Overexpression of MMSET promoted tumor formation, and MMSET-knockdown HEC-1 cells generated smaller tumors (Figure 1I and 1J), suggesting that MMSET contributes to EC tumor growth *in vivo*.

### Twist1 mediates MMSET-induced sphere formation and invasion

MMSET acts as an upstream regulator of Twist1 to induce EMT and invasion in prostate cancer [11]. To test whether the tumor-promoting effects of MMSET are mediated by Twist1 in EC, we examined the protein expression of Twist1 and its downstream effectors Vimentin and E-cadherin in EC cells following either MMSET overexpression or knockdown. Western blotting analysis revealed that Twist1 and Vimentin levels were increased, while the expression of E-cadherin was decreased in Ishikawa cells after transient MMSET overexpression (Figure 1H). Consistent with these results, knockdown of MMSET via siRNA reduced Twist1 and Vimentin expression, whereas induced E-cadherin expression in HEC-1 cells (Figure 1H).

**Figure 2:**
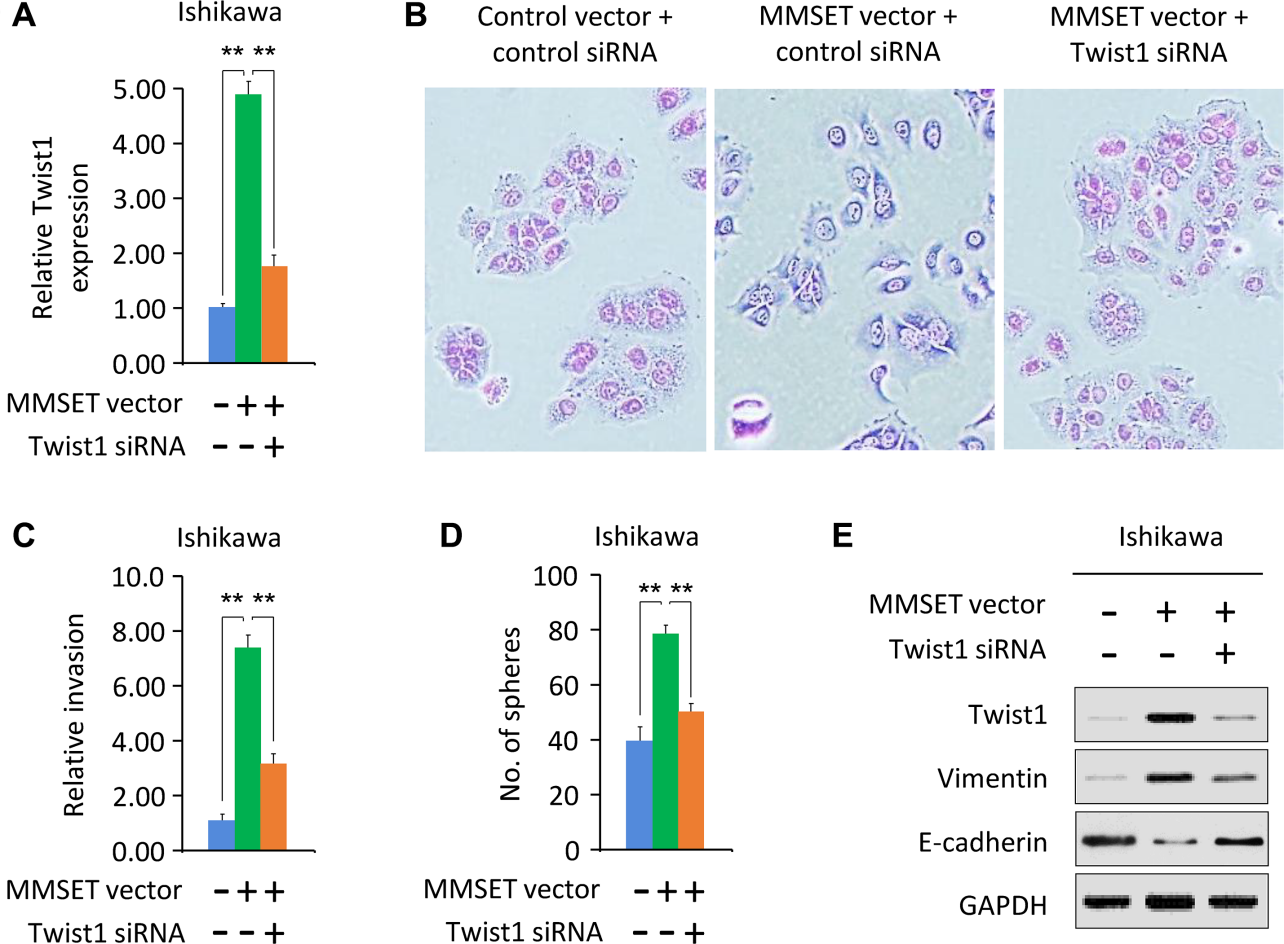
Twist1 mediates MMSET-induced sphere formation and invasion (**A**) Ishikawa cells were transfected with control vector or MMSET-expressing vector, together with siRNA targeting MMSET or control siRNA. Relative *Twist1* expression was analyzed using qPCR. (**B**) Morphological appearance of cells described in (A) was analyzed by microscopy. (**C** and **D**) Invasion (C) and sphere formation (D) of cells described in (A) was determined using transwell invasion and sphere formation assay. (**E**) Western blotting analysis of indicated proteins in cells described in (A). ^**^P < 0.01.

To evaluate whether MMSET promotes EC cell invasion and sphere formation by regulating Twist1 expression, we co-transfected MMSET expression vector together with Twist1-specific siRNA into Ishikawa cells. We found that Twist1 mRNA expression was increased in response to upregulation of MMSET, but was decreased after knockdown of Twist1 (Figure 2A). The scattered morphology induced by MMSET overexpression was partially reverted by Twist1 silencing, resulting in a more epithelial-like appearance (Figure 2B). The downregulation of Twist1 significantly reduced MMSET-mediated invasion and sphere formation (Figure 2C and 2D). MMSET-induced Vimentin expression in Ishikawa cells was decreased following Twist1 silencing, and MMSET-mediated E-cadherin repression was reversed upon Twist1 siRNA transfection (Figure 2E). These data suggest the oncogenic role of MMSET in EC cells, and that Twist1 is required for MMSET-induced sphere formation, EMT and invasion.

### Elevated MMSET expression predicts poor survival in EC

**Figure 3:**
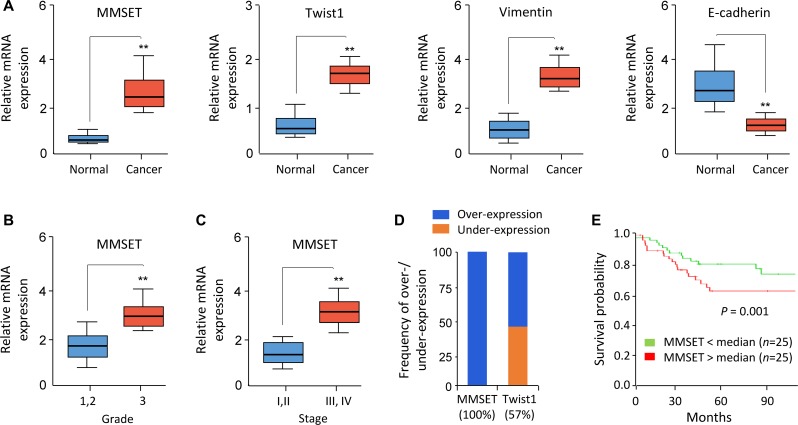
Elevated MMSET expression predicts poor survival in EC (**A**) Relative mRNA expression of *MMSET*, *Twist1*, *Vimentin* and *E-cadherin* in 50 matched human normal endometrial tissues and EC tissues. (**B** and **C**) Relative *MMSET* mRNA levels in EC samples were categorized based on tumor grade (B) and stage (C). (**D**) Meta-analysis of *MMSET* and *Twist1* mRNA in EC samples and adjacent normal tissues from the BioExpress database. (**E**) Kaplan–Meier overall survival curves for high and low *MMSET* mRNA expression in EC patients. ^**^P < 0.01.

To further assess the clinical correlation between MMSET and Twist1, we investigated the mRNA expression level of *MMSET*, *Twist1*, *Vimentin* and *E-cadherin* in 50 pairs of EC and adjacent normal endometrial tissues using qPCR assay. EC tissue displayed high levels of *MMSET*, *Twist1* and *Vimentin*, but low levels of *E-cadherin* (Figure 3A). Importantly, increased *MMSET* expression in EC correlated with higher tumor grade and advanced tumor stage (Figure 3B and 3C). In addition, a meta-analysis of EC samples and adjacent normal samples from published RNA sequencing studies in BioXpress database revealed that *MMSET* and *Twist1* were overexpressed in 100% and 57% of EC samples compared with their paired normal samples, respectively (Figure 3D). To further evaluate the potential correlation of *MMSET* expression with patient outcome, 50 EC patients were divided into two groups based on the median value of *MMSET* mRNA expression: high MMSET group (*n =* 25) or low MMSET group (*n =* 25). Kaplan-Meier analysis revealed that increased *MMSET* mRNA expression was significantly correlated with poor overall survival in EC (Figure 3E). Taken together, MMSET overexpression enhances EC metastatic capability by upregulating Twist1, and overexpression of MMSET represents a prognostic factor in EC outcome.

### miR-34a, miR-424 and miR-513 inhibit EMT, invasion and the sphere-forming ability of EC cells through targeting MMSET

**Figure 4:**
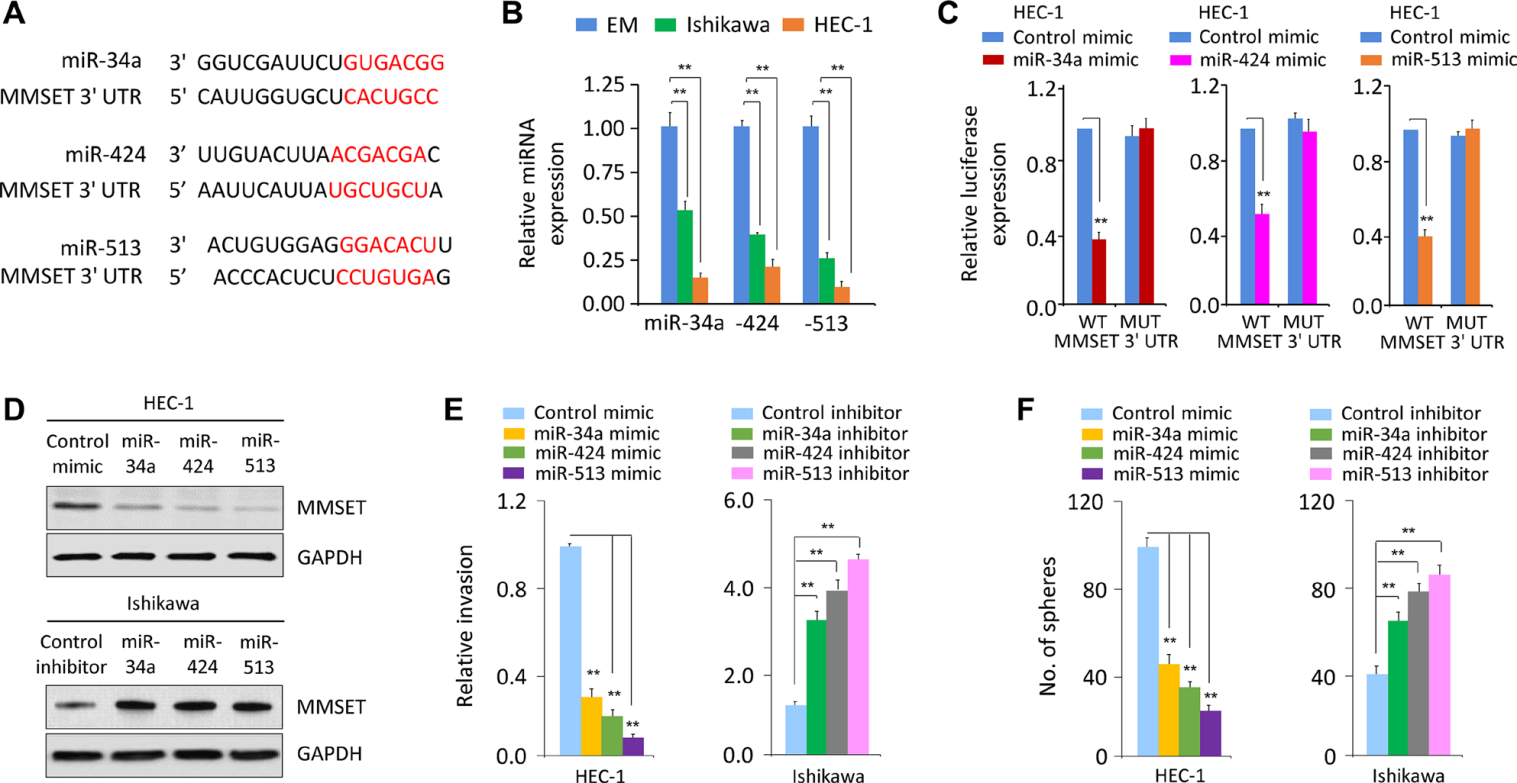
miR-34a, miR-424 and miR-513 directly target MMSET 3′-UTR to downregulate MMSET expression in EC cells (**A**) Alignment of miR-34a, miR-424 and miR-513 and their corresponding complementary binding sequences in *MMSET* 3′-UTR by bioinformatics algorithms. (**B**) Relative miR-34a/miR-424/miR-513 levels in EC cell lines and EM cells. (**C**) HEC-1 cells were transfected with reporter constructs containing either wild-type (WT) MMSET, or MMSET 3′-UTR with mutation (MUT), along with miR-34a/miR-424/miR-513 mimic or negative control mimic, respectively. Relative luciferase activity was measured. (**D**) Western blotting analysis of MMSET expression in EC cells after overexpression or knockdown of miR-34a/miR-424/miR-513. (**E** and **F**) Cell invasion (E) and sphere formation (F) of EC cells after overexpression or knockdown of miR-34a/miR-424/miR-513. ^**^P < 0.01.

We asked whether miRNA inhibition is responsible for MMSET overexpression observed in EC. First, we performed *in silico* analysis using target prediction databases (TargetScan and miRDB) to identify potential miRNAs that are computationally predicted as regulators of MMSET. Then, these predicted miRNAs were overlapped with a set of miRNAs that are significantly repressed in highly invasive EC cells [16], leading to the identification of 3 miRNAs (miR-34a, miR-424 and miR-513) (Figure 4A).

Using qPCR analysis, we found that invasive HEC-1 cells had very low miR-34a/miR-424/miR-513 expression compared with less invasive Ishikawa cells (Figure 4B), indicating that the levels of miR-34a/miR-424/miR-513 were inversely correlated with MMSET expression in EC cells (Figure 1A). To test whether MMSET is directly regulated by miR-34a/miR-424/miR-513, we performed luciferase reporter assays by transfecting the reporter vector containing the full-length 3′-UTR of human *MMSET* into HEC-1 cells, together with miR-34a, miR-424, miR-513 or control mimic, respectively. Ectopic expression of miR-34a/miR-424/miR-513 in HEC-1 cells resulted in a significant decrease in the relative luciferase activity of *MMSET* 3′-UTR, but there was no inhibition of luciferase activity when the cells were transfected with miR-34a/miR-424/miR-513 mimic and mutant *MMSET* 3′-UTR (Figure 4C). Consistently, our western blot data showed that transfection of miR-34a/miR-424/miR-513 reduced MMSET expression in HEC-1 cells, whereas transfection of miR-34a/miR-424/miR-513 inhibitor enhanced MMSET expression in Ishikawa cells (Figure 4D), suggesting that miR-34a/miR-424/miR-513 directly repress MMSET.

Importantly, overexpression of miR-34a/miR-424/miR-513 inhibited invasion and sphere formation of HEC-1 cells (Figure 4E and 4F). However, Ishikawa cells transfected with miR-34a/miR-424/miR-513 inhibitor exhibited significantly increased cell invasion and sphere formation (Figure 4E and 4F). These observations suggest that miR-34a/miR-424/miR-513 are suppressors of EC cell invasion and sphere formation.

**Figure 5:**
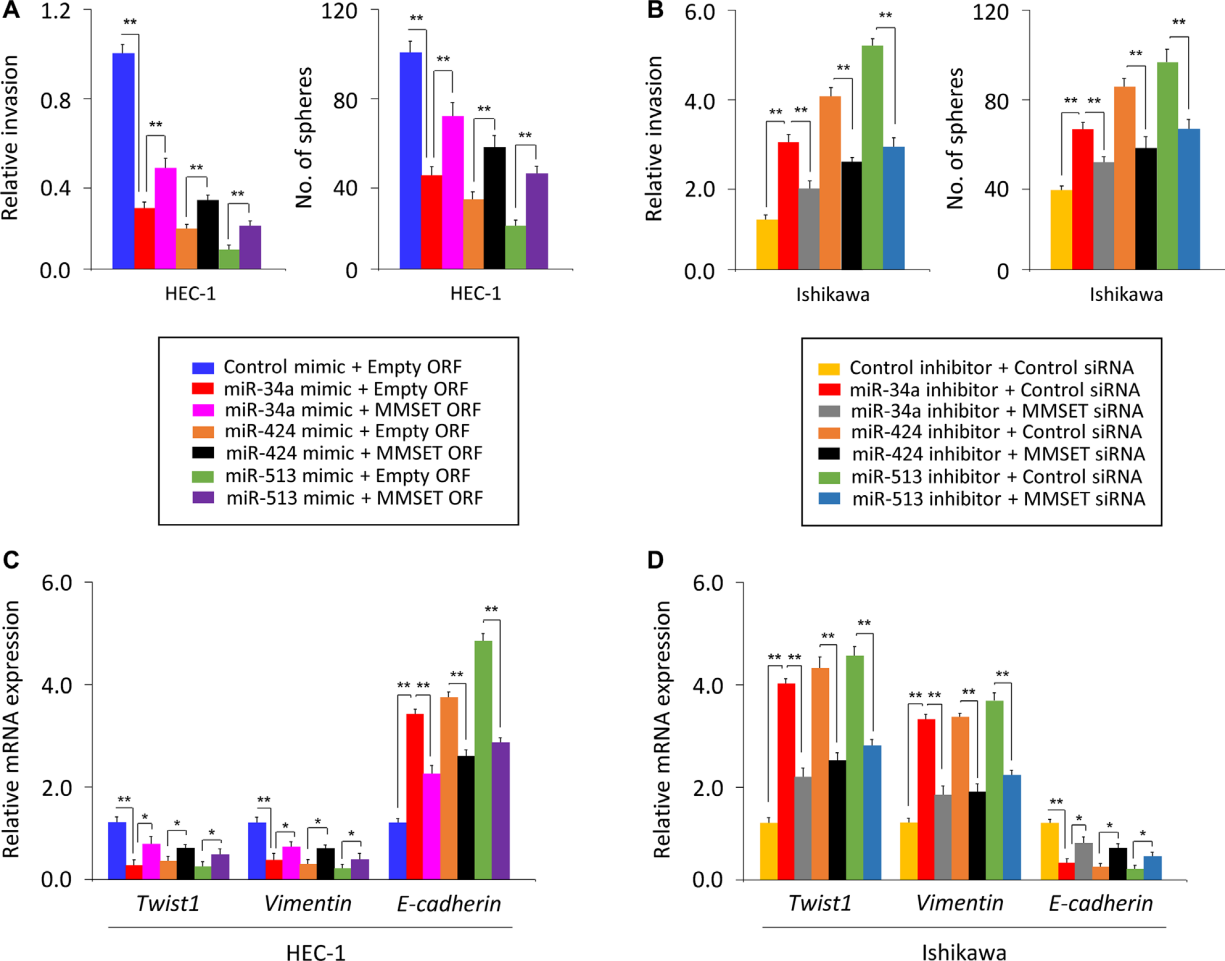
miR-34a, miR-424 and miR-513 inhibit EMT, invasion and the sphere-forming ability of EC cells through targeting MMSET (**A**) miR-34a/miR-424/miR-513 mimic or control mimic was co-transfected into HEC-1 cells, together with (or without) MMSET cDNA vector lacking the 3′-UTR region. (**B**) miR-34a/miR-424/miR-513 inhibitor or control inhibitor was co-transfected into Ishikawa cells, together with (or without) MMSET siRNA. Cell invasion and sphere formation assays were performed. (**C** and **D**) qPCR analysis of indicated genes in HEC-1 (C) and Ishikawa (D) cells treated as described above. ^*^P < 0.05, ^**^P < 0.01.

To further validate the above observations, we investigated whether transient over-expression of a MMSET open reading frame (ORF) could reverse the inhibitory effects of miR-34a/miR-424/miR-513 mimic on EC cell invasion and sphere formation, or whether silencing of MMST with specific siRNA could repress the miR-34a/miR-424/miR-513 inhibitor-induced EC cell invasion and sphere formation. The overexpression of MMSET ORF in HEC-1 cells partially rescued miR-34a/miR-424/miR-513 mimic-suppressed invasion and sphere formation (Figure 5A). Moreover, the miR-34a/miR-424/miR-513 inhibitor-induced Ishikawa cell invasion and sphere formation were significantly reduced by MMSET siRNA (Figure 5B).

We measured *Twist1*, *Vimentin* or *E-cadherin* mRNA by qPCR assay, and found that rescuing MMSET expression with MMSET ORF in the presence of miR-34a/miR-424/miR-513 mimic resulted in up-regulation of *Twist1* and *Vimentin* and downregulation of *E-cadherin* (Figure 5C). Consistently, inhibiting MMSET expression using siRNA in Ishikawa cells transfected with miR-34a/miR-424/miR-513 inhibitor reduced the expression of *Twist1* and *Vimentin* but elevated the expression of *E-cadherin* (Figure 5D). Taken together, our data suggest that miR-34a/miR-424/miR-513 inhibits the invasive and stem cell-like properties of EC cells by suppressing MMSET expression via interacting with its 3′-UTR.

### miR-34a/miR-424/miR-513 repression was associated with poorer prognosis of EC patients

**Figure 6:**
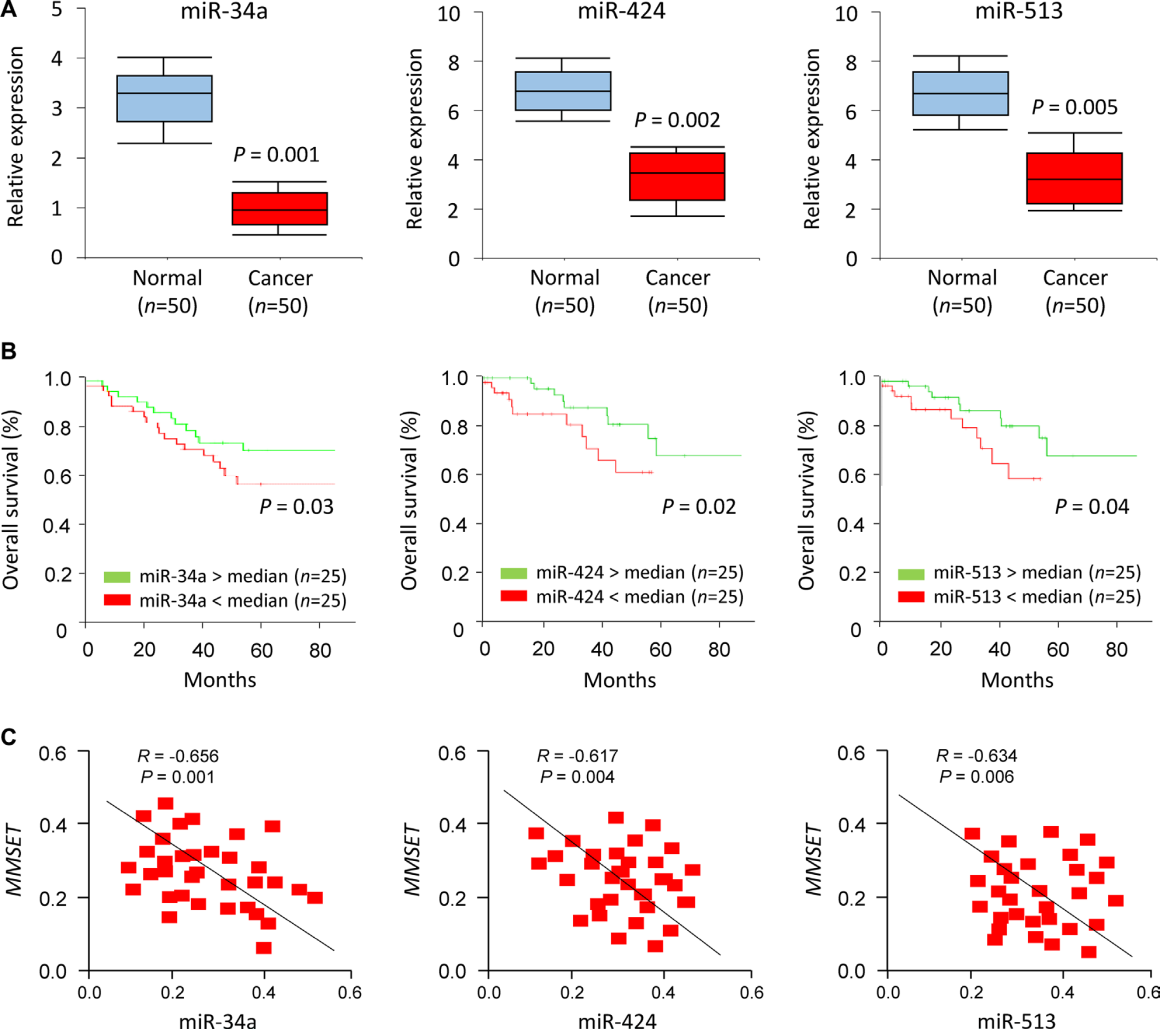
miR-34a/miR-424/miR-513 repression was associated with poorer prognosis of EC patients (**A**) Relative miR-34a/miR-424/miR-513 expression in EC tissues and matched adjacent normal endometrial tissues. (**B**) Kaplan–Meier overall survival curves for high and low miR-34a/miR-424/miR-513 expression in EC patients. (**C**) Correlation of expression between miR-34a/miR-424/miR-513 expression levels and PD-L1 mRNA expression in EC patients.

To address the relevance of miR-34a/miR-424/miR-513 expression to human EC, we examined the levels of these miRNAs in primary ECs using qPCR analysis. Significant reduction in miR-34a/miR-424/miR-513 levels were apparent in EC samples compared to adjacent normal endometrial tissues (Figure 6A). To determine if reduced miR-34a/miR-424/miR-513 expression is associated with any change in survival probability, we compared Kaplan-Meier plots for high and low expression of miR-34a/miR-424/miR-513. For a set of 50 patients with EC, reduced expression of miR-34a/miR-424/miR-513 was significantly associated with a poorer prognosis of EC patients (Figure 6B), To explore whether the miR-34a/miR-424/miR-513-MMSET axis is clinically relevant, we assessed the correlation between the expression of miR-34a/miR-424/miR-513 and MMSET in EC tissues using qPCRs. We detected a significant negative association between miR-34a/miR-424/miR-513 and MMSET mRNA expression (Figure 6C). Taken together, these results supported an existence of the miR-34a/miR-424/miR-513-MMSET axis in human EC.

### Pharmacological inhibition of MMSET via BIX-01294 inhibits EC cell invasion and sphere formation

**Figure 7:**
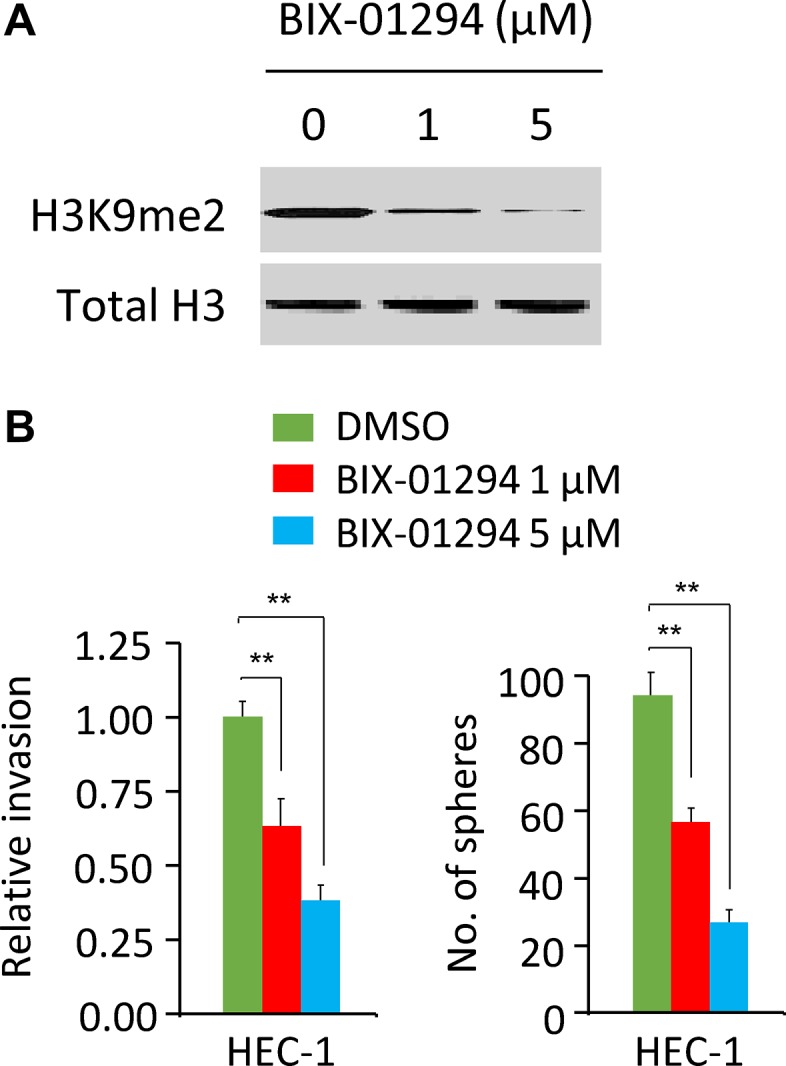
BIX-01294 treatment suppresses cell invasion and sphere formation in EC cells (**A**) Western blotting analysis of H3K9me2 and total histone H3 (loading control) in HEC-1 cells treated with 0, 1 or 5 μM BIX-01294. (**B**) Cell invasion assays and sphere formation assays were carried out in HEC-1 cells treated with 0, 1 or 5 μM BIX-01294. ^**^P < 0.01.

BIX-01294, a selective and specific inhibitor of MMSET [13], has been shown to impair cell growth and sphere formation of hepatocellular carcinoma cells [14]. To study whether pharmacological inhibition of MMSET activity can inhibit EC cell invasion and sphere formation, we treated HEC-1 cells with various concentrations (0, 1, 5 μM) of BIX-01294. Our results suggested that as low as 1 μM BIX-01294 treatment could reduce the level of H3K9me2 in HEC-1 cells, and BIX-01294 inhibited the invasion and sphere formation of HEC-1 cells in a dose-dependent manner (Figure 7), supporting that pharmacological inhibition of MMSET via BIX-01294 can suppress EC cell invasion and sphere formation.

## DISCUSSION

The histone methyltransferase MMSET is frequently overexpressed in a wide range of cancer types and plays an essential role in tumorigenesis and metastasis [9, 15, 16]. In this study, we have uncovered for the first time that overexpression of MMSET contributes to the stem cell-like and invasive properties of EC cells by upregulating Twist1, and specific inhibition of MMSET activity by BIX-01294 inhibits EC cell invasion and sphere formation. We also found that repression of miR-34a/miR-424/miR-513 upregulates MMSET levels, thus promoting the invasion and sphere-forming ability of EC cells.

Multiple independent studies have demonstrated that MMSET is involved in regulating cancer tumorigenicity and metastasis [17–19]. For example, MMSET promotes cell cycle progression through direct transcriptional upregulation of NEK7, which is a downstream target gene of MMSET in squamous cell carcinomas of the head and neck [17]. MMSET induces multiple myeloma cell growth through silencing of miR-126^*^ and the subsequent overexpression of c-MYC, a target of miR-126^*^ [18]. Moreover, MMSET interacts with NF-κB directly to activate the expression of NF-κB target genes (including interleukin-6, vascular endothelial growth factor A, cyclin D, Bcl-2 and Survivin) in prostate cancer cells [19]. Further investigation of the mechanism by which MMSET activates Twist1 or other EMT-associated molecules in EC cells is worthwhile.

miR-26a, miR-31 and miR-203 were shown to inhibit MMSET expression in prostate cancer [20]. However, the critical miRNAs that function as tumor suppressors by inhibiting MMSET in EC remain largely unknown. In this study, we identified miR-34a/miR-424/miR-513 that could directly regulate MMSET expression in EC cells. Our results demonstrated that the expression of miR-34a/miR-424/miR-513 is frequently lost in human EC tissues, and ectopic miR-34a/miR-424/miR-513 expression reduced EC cell sphere formation and invasion.

miR-34a and miR-424 are reduced and thought to be tumor suppressors in EC [21, 22]. Consistent with these studies, we found low levels of miR-34a and miR-424 in EC cell lines and EC patient tissues, and these low expressions correlated with a poorer prognosis in EC. Furthermore, in the present work, we provide new evidence that miR-513 can suppress EC cell sphere formation and invasiveness by targeting MMSET.

In conclusion, our results demonstrate that MMSET exerts tumor-promoting effects in EC cells, and the loss of miR-34a, miR-424 and miR-513 enhances MMSET expression in EC. This study also suggests that pharmacological inhibition of MMSET with BIX-01294 might be a promising therapeutic approach for EC.

## MATERIALS AND METHODS

### Cell culture and reagents

The human EC cell lines Ishikawa and HEC-1 were grown in DMEM/F12 medium (Invitrogen, Carlsbad, CA) supplemented with 10% fetal bovine serum (FBS, Invitrogen, Carlsbad, CA). All fresh cell lines were purchased from the JCRB Cell Bank (Osaka, Japan). All the cell lines used in this study were within six passages after receipt. The cell lines were not authenticated as they came from national repositories. These cell lines were routinely tested by PCR for mycoplasma contamination by using the following primers: Myco_fw1: 5′-ACACCATGGGAGCTGGTAAT-3′, Myco_rev1: 5′-CTTCATCGACTTTCAGACCCAAGGCAT-3′. The immortalized human endometrial epithelial EM cell line was generated and extensively characterized by Satoru Kyo (Shimane University, Japan) [23]. BIX-01294 was purchased from Sigma-Aldrich (St. Louis, MO). When reaching 50% confluence, HEC-1 cells were treated with different concentrations of BIX-01294 for 24 hours.

### Transient transfection

EC cells (50% confluence) were transfected with miRNA mimics and miRNA inhibitors for miR-34a, miR-424 and miR-513 (40 nM, Ambion, Austin, TX), siRNAs against MMSET or Twist1 (5 nM, Ambion, Austin, TX) and the expression vector for MMSET (OriGene, Rockville, MD) using Lipofectamine 3000 (Invitrogen, Carlsbad, CA) according to the manufacturer’s instructions. After Forty-eight hours, the cells were used for RNA extraction, protein extraction, transwell invasion assay and tumor sphere formation assay.

### Real-time quantitative RT-PCR (qPCR)

Total RNA was extracted using TRIZOL reagent (Invitrogen, Carlsbad, CA) according to the manufacturer’s instructions. cDNA was synthesized using the PrimeScript RT reagent kit (Takara, Japan). Real-time PCR was conducted by using ABI PRISM 7000 Sequence Detection System (Applied Biosystems). The primer sequences for mRNA detection were purchased from Applied Biosystems. miR-34a, miR-424 and miR-513 expression was measured using the NCode miRNA qRT-PCR analysis (Invitrogen, Carlsbad, CA) following manufacturer-recommended protocols. Forward primers are the exact sequences of the mature miR-34a, miR-424 and miR-513. For normalization, *GAPDH* and U6 were used as endogenous controls to normalize mRNA and miRNA expression levels. Results were expressed as fold change in expression levels relative to those of respective controls, the average value of which was taken as 1.

### Tumor sphere formation assay

EC cells (1000 cells) were grown in serum-free DMEM/F12 medium supplemented with N2 plus media supplement (Invitrogen), 20 ng/ml epidermal growth factor (Invitrogen, Carlsbad, CA), 20 ng/ml basic fibroblast growth factor (Invitrogen, Carlsbad, CA) and 4 mg/ml heparin (Sigma-Aldrich, St. Louis, MO) in 24-well ultra-low attachment plate (Corning, NY) for 14 days. Then the number of floating spheres larger than 50 μm was counted.

### Transwell invasion assay

Transwell invasion assay was performed as previously reported [24–26]. EC cells were grown to 50% confluence and transfected as indicated. After 24 hours, cells (5 × 10^4^) were seeded into the upper chamber of Boyden chambers coated with Matrigel in 24-well plate with 8.0 µm pores (Corning, NY). Complete medium with 10% FBS (700 μl) served as a chemoattractant in the bottom chamber. After incubation for 24 hours, the cells on the Matrigel side of the inserts were removed by cotton swab. The inserts were fixed in methanol and stained with Giemsa. The number of invaded cells attached to the other side of the insert was counted under a microscope. Relative invasion was expressed as the fold change relative to respective control.

### Western blotting

A total of 30 µg protein was separated by 10% SDS-PAGE, and membranes were incubated with primary antibodies against human MMSET (HPA015801, Sigma-Aldrich, St. Louis, MO), Twist1 (ab50887, Abcam, Cambridge, MA), Vimentin (A01189, GenScript, Edison, NJ), E-cadherin (A01589, GenScript, Edison, NJ), H3K9me2 (4658, Cell Signaling, Danvers, MA), total histone H3 (4499, Cell Signaling, Danvers, MA) and GAPDH (sc-47778, Santa Cruz, Santa Cruz, CA), followed by HRP-conjugated secondary antibody and developed using the ECL reagent (Amersham, Poole, UK).

### Luciferase reporter assay

HEC-1 cells were seeded on 24-well tissue culture plates and allowed to adhere for 24 hours. The *MMSET* 3′-UTR luciferase vector was obtained from by RiboBio Co., Ltd. (Guangzhou, China). Mutation in the miR-34a, miR-424 or miR-513-binding sequence was generated by using the QuickChange Mutagenesis Kit (Stratagene, La Jolla, CA). Firefly luciferase reporter plasmid (100 ng) plus Renilla luciferase vector (10 ng), together with miR-34a/miR-424/miR-513 mimic or the negative control mimic, were transfected into HEC-1 cells using Lipofectamine 3000 (Invitrogen, Carlsbad, CA). Firefly Luciferase and Renilla luciferase signals were measured 48 hours after transfection using a Dual-Luciferase Reporter Assay (Promega, Madison, WI) according to the manufacturer’s instructions. The relative firefly luciferase activities were normalized against the Renilla luciferase activities.

### Tumor xenograft experiments

Experiments involving mice were performed under protocols approved by the Animal Care and Use Committee of Sun Yat-Sen University Cancer Center. Four-week-old of female BALB/c mice were acquired from Shanghai Laboratory Animal Center (Chinese Academy of Sciences, Shanghai, China). The tumor generation assay *in vivo* was performed as previously described [24]. In brief, EC cells were transfected with MMSET expression vector, MMSET siRNA or their respective controls, respectively. 48 hours after transfection, 1 × 10^6^ cells were suspended in phosphate-buffered saline and then injected subcutaneously into nude mice. Tumor length and width were measured using calipers, and tumor volume was calculated using the formula: tumor volume = length × width^2^ × 0.5.

### Patients and tissue samples

Fresh human EC tissues and the corresponding adjacent normal endometrial samples were collected from 50 patients who underwent surgical resection at the Sun Yat-Sen University Cancer Center, China. This study was approved by the Clinical Research Ethnics Committee of Sun Yat-Sen University. Informed consent was obtained from all patients. All experiments were performed in accordance with relevant guidelines and regulations. All tissue specimens were snap-frozen immediately in liquid nitrogen after harvesting and stored at −80°C.

### Statistical analysis

The results were reported as mean ± SEMs of at least three independent experiments. Statistical analysis was conducted using 2-tailed Student’s *t*-test or 1-way ANOVA. Differences in mRNA or miRNA expression between EC tissues and normal endometrial tissues were evaluated using the Mann-Whitney *U*-test. The log-rank test was used to compare differences between the survival curves. *P <* 0.05 was considered statistically significant.
